# Utility of Tei Index, carotid IMT, and crouse score in coronary artery calcification assessment and MACCE prediction in elderly patients

**DOI:** 10.3389/fcvm.2026.1781737

**Published:** 2026-05-21

**Authors:** Haifeng Zang, Peiliang Cheng, Caifei Jin

**Affiliations:** 1 Ultrasound Department. South Taihu Hospital Affiliated To Huzhou College, Huzhou, Zhejiang Province, China; 2 Health Management Center, Zhejiang Provincial Hospital of Dermatology, Huzhou, Zhejiang, China

**Keywords:** carotid intima-media thickness, comprehensive myocardial index, coronary artery calcification, crouse plaque score, MACCE

## Abstract

**Objective:**

Current study aims to evaluate the diagnostic and prognostic value of integrating myocardial performance index (Tei index), carotid intima-media thickness (IMT), and Crouse plaque score in coronary artery calcification (CAC) severity and major adverse cardiovascular and cerebrovascular events (MACCE) recurrence among elderly coronary heart disease (CHD) patients

**Methods:**

A retrospective study included 98 elderly CHD patients (2019–2022) from South Taihu Hospital. Coronary artery calcification score (CACS) was assessed, with severe calcification defined as single-vessel CACS ≥ 100. Participants were stratified into severe calcification (*n* = 45) and control (CACS < 100, *n* = 53) groups. Baseline characteristics, Tei index, IMT, and Crouse score were compared. Multivariate logistic regression identified CAC risk factors. Spearman analysis assessed correlations between CACS and biomarkers. Receiver operating characteristic (ROC) curves evaluated combined diagnostic performance. Kaplan–Meier survival analysis predicted MACCE recurrence using optimal thresholds from ROC.

**Results:**

The severe calcification group exhibited elevated Tei index [0.58 (0.51, 0.72) vs. 0.44 (0.35, 0.52)], IMT [1.11 (0.89, 1.26) mm vs. 0.72 (0.62, 0.80) mm], and Crouse score [1.83 (1.04, 2.56) vs. 0.90 (0.77, 1.09)] compared to controls (all *p* < 0.001). These parameters independently predicted severe CAC (OR = 2.634–73.061, *p* < 0.05) and correlated positively with number of stenosed coronary vessel (*r* = 0.429–0.453, *p* < 0.01). Combined ROC analysis demonstrated superior diagnostic accuracy (AUC = 0.831, 95% CI:0.740–0.923) vs. individual biomarkers (AUC = 0.778–0.792). High Tei index (≥0.50), IMT (≥0.86 mm), and Crouse score (≥1.21) predicted increased MACCE risk (log-rank *p* < 0.001).

**Conclusion:**

The synergistic application of Tei index, carotid IMT, and Crouse score enhances CAC diagnosis precision and stratifies MACCE recurrence risk in elderly CHD patients, offering a noninvasive strategy for postoperative risk management.

## 1 Introduction

Cardiovascular diseases (CVDs) pose a major global health burden, associated with high mortality and morbidity rates (1). Among them, coronary heart disease (CHD) is the most prevalent (2). With the rapid aging of the global population, CHD has emerged as a pressing health concern (3). According to a 2022 statistical study, China harbors 330 million individuals with CVD, including 11.39 million with CHD (4). Notably, CHD predominantly affects the elderly, underscoring the importance of early identification in this population (5). Coronary artery calcification (CAC), a robust biomarker of coronary atherosclerosis, is closely associated with traditional CVD risk factors, recurrent cardiovascular events post-revascularization, and future cardiovascular events (6). The prevalence of CAC increases with age, exceeding 90% in men and 67% in women over 70 years old (7). While invasive coronary angiography (ICA) remains the gold standard for CHD diagnosis, its invasive nature and reduced tolerability among the elderly population limit its applicability. Alternatively, coronary computed tomography angiography (CCTA) with fractional flow reserve derived from CT (CT-FFR) offers a non-invasive, convenient, and highly accurate means of assessing coronary hemodynamics (8, 9). However, CAC-related blooming artifacts and partial volume effects can significantly compromise CCTA's diagnostic accuracy and specificity for CHD (10). Similarly, CT-FFR can be affected by severe CAC, reducing the precision of results (11). Accurate identification of severe CAC is thus pivotal for early CHD diagnosis and treatment.

In recent years, echocardiography has gained prominence due to its simplicity, non-invasiveness, safety, and reproducibility, thereby enhancing diagnostic accuracy and informing subsequent treatment and intervention strategies for CHD (12). Echocardiography facilitates the visualization of abnormal ventricular wall motion and the assessment of myocardial ischemia and its extent, which are indicative of coronary involvement (13). The Tei index, a novel metric that evaluates cardiac function by integrating systolic and diastolic time intervals, is characterized by its simplicity, reliability, and independence from left ventricular geometry, blood pressure, or heart rate, effectively distinguishing between impaired and normal cardiac function (14, 15). The carotid artery, a critical conduit between the heart and brain, can be assessed via carotid ultrasound, which not only reveals carotid disease but also evaluates the risk of coronary atherosclerosis (16). Numerous studies have established a strong correlation between coronary and carotid atherosclerosis (17). Carotid ultrasound measurements, such as the Crouse plaque score and intima-media thickness (IMT), provide significant diagnostic value for coronary artery disease (18). However, the combined application of Tei index, carotid IMT, and Crouse plaque score in severe CAC diagnosis remains unexplored.

As the population ages and diabetes and chronic kidney disease prevalence rises, severe CAC lesions are increasingly encountered in patients undergoing percutaneous coronary intervention (PCI) (19, 20). Intravascular ultrasound (IVUS) and optical coherence tomography (OCT) play pivotal roles in diagnosing and guiding treatment strategies for severe calcification (19, 21). While CAC scores have been used to assess CHD risk and prognosis (22–24), with higher scores correlating with increased cardiovascular event risk (25, 26), research on prognosis in elderly patients with severe CAC is scarce. Identifying suitable surrogate markers for severe CAC is crucial for diagnosis, treatment, and prognosis in this population. This study aims to analyze the screening value of echocardiography combined with carotid ultrasound in elderly patients with severe CAC and explore the risk of recurrent adverse cardiovascular events post-intervention.

## 2 Materials and methods

### 2.1 Study population

Ninety-eight elderly CHD patients aged ≥60 years (56 males and 42 females) hospitalized in the Department of Cardiology, South Taihu Hospital, from January 2019 to December 2022, were enrolled. Patients were stratified into a focal severe coronary calcification group (CACS ≥ 100, *n* = 45) and a control group (CACS < 100, *n* = 53) based on coronary artery calcification scores (CACS). Inclusion criteria: 1) All patients underwent echocardiography and carotid ultrasound with complete imaging data; 2) CCTA and Agatston method were used to calculate calcification scores during hospitalization; 3) All patients with severe CHD underwent percutaneous coronary intervention (PCI), also known as coronary stent implantation or drug-eluting balloon therapy; 4) Patients provided informed consent. (Note: The inclusion criterion regarding informed consent is typically included in studies involving human subjects but can be tailored to specific research protocols and ethical requirements.) Exclusion criteria: 1) Malignancies; 2) Concomitant liver/gallbladder diseases, renal dysfunction, cerebrovascular diseases, ventricular arrhythmias, or heart failure; 3) Prior revascularization procedures; 4) Severe infections; 5) Severe contrast media allergies; 6) Follow-up duration of less than one year or loss to follow-up.

### 2.2 Methods

#### 2.2.1 Echocardiography

Echocardiographic evaluations were performed using a color Doppler ultrasound system (GE Vivid E95, Wauwatosa, WI, USA). Patients were instructed to lie in a left lateral decubitus position with the chest area fully exposed. The transducer frequency was set at 2–5 MHz, and the probe was sequentially scanned from the parasternal and apical regions. Subsequently, patients were shifted to a supine position for scanning the heart and major vessels, with measurements of the Tei index.

#### 2.2.2 Carotid ultrasound

The transducer frequency of the Doppler ultrasound system (GE Vivid E95, USA) was adjusted to 3–12 MHz. Patients were positioned supine with their necks fully exposed. Multiplanar scanning was performed to obtain transverse and longitudinal views of both carotid arteries. The intima-media thickness (IMT) at the bifurcation, the origins of the external and internal carotid arteries, and the common carotid arteries were measured, with the maximum value considered representative of carotid atherosclerosis. Plaque formation was analyzed and scored based on the maximum thickness of isolated atherosclerotic plaques in the ipsilateral carotid artery, summing the scores from both sides to obtain the total plaque score, disregarding the length of each plaque (27).

#### 2.2.3 Coronary computed tomography angiography (CCTA)

CCTA was conducted using a second-generation dual-source computed tomography scanner (GE Optima 670, Waukesha, WI, USA). Briefly, the scan parameters included a gantry rotation time of 280 ms, detector collimation of 2 mm × 64 mm, slice thickness of 0.6 mm, and pitch of 3.4. Patients underwent respiratory training and received sublingual nitroglycerin (0.5 mg, Beijing Yimin Pharmaceutical Co., Ltd.) 3 min prior to the scan. A bolus of iodinated contrast agent (Iopromide 37 g/100 mL, Shanghai Bracco Sine Pharmaceutical Corp., Ltd.) was injected intravenously at a rate of 4.5–5.0 mL/s. Scanning was triggered when the CT threshold in the region of interest (ROI) of the ascending aorta reached 100 HU. Image quality control for CCTA was assessed using a 5-point Likert scale (28).

#### 2.2.4 Follow-up

All patients with significant coronary artery calcification (CAC) underwent revascularization (RA). Postoperative follow-up was conducted through outpatient visits or telephone interviews. The follow-up ended on December 31, 2022. The primary endpoint was major adverse cardiovascular and cerebrovascular events (MACCE) during the follow-up period, defined as all-cause death, non-fatal myocardial infarction, target vessel revascularization, and non-fatal stroke.

### 2.3 Statistical analysis

Statistical analysis was performed using SPSS 27.0. Nonparametric tests were employed to assess data normality. Normally distributed continuous variables were presented as Mean ± SD and compared using independent *t*-tests. Non-normally distributed data were expressed as medians (interquartile ranges) and compared with the Kolmogorov–Smirnov test. Categorical variables were reported as percentages and analyzed using chi-square tests. One-way ANOVA was applied for comparisons among multiple groups, with LSD tests for *post hoc* pairwise comparisons. Univariate and multivariate binary logistic regression analyses were conducted to identify independent risk factors for significant CAC. Spearman's correlation was used to analyze associations between variables. The diagnostic performance of different modalities in significant CAC was evaluated using receiver operating characteristic (ROC) curves. Univariate and multivariate Cox proportional hazards regression analyses, together with Kaplan–Meier survival curves, were employed to assess the risk of cardiovascular events and predict postoperative outcomes in patients with significant CAC post-RA. Statistical significance was set at *P* < 0.05.

## 3 Results

### 3.1 Baseline characteristics

Based on the inclusion and exclusion criteria, 98 patients with coronary artery disease (CAD) were included. Patients were stratified into a significant calcification group (CACS ≥ 100, *n* = 45) and a control group (CACS < 100, *n* = 53). As shown in Table 1, the mean age of patients in the significant calcification group was 69 (65, 75) years, with 24 (53.33%) males. The control group had a mean age of 71 (68, 75) years, with 32 (60.38%) males. No significant differences were observed in age or gender distribution between the two groups (both *P* > 0.05). Furthermore, the proportions of patients with traditional cardiovascular risk factors such as smoking, alcohol consumption, hypertension, diabetes, or hyperlipidemia were 30.61%, 27.55%, 31.63%, 7.14%, and 60.20%, respectively, with no significant differences between the groups (all *P* > 0.05). Similarly, triglyceride (TG), total cholesterol (TC), high-density lipoprotein cholesterol (HDL-C), and low-density lipoprotein cholesterol (LDL-C) levels were comparable between the two groups (both *P* > 0.05). These results indicated that the baseline characteristics were comparable, allowing for further analysis of Tei index, carotid IMT, and Crouse plaque score, which were significantly higher in the significant calcification group compared to the control group (all *P* < 0.001). *post-hoc* power analysis for these comparisons revealed a statistical power exceeding 90% for detecting the observed differences in Tei index and IMT, and 85% for the Crouse score.

**Table 1:** Comparison of general data of patients with coronary heart disease.

Parameters	CACS ≥ 100 (*n* = 45)	CACS <100 (*n* = 53)	*t*/*Z*/*X*^2^	*P*
Age (year)	69 (65, 75)	71 (68, 75)	0.666	0.767
Gender (Male/female)	24/21	32/21	0.493	0.483
BMI (kg/m^2^)	25.37 (23.29, 27.06)	24.49 (22.56, 26.53)	0.753	0.622
Smoking history (%)	15 (33.33)	15 (28.30)）	0.290	0.590
Drinking history (%)	13 (28.89)	14 (26.42)	0.075	0.785
Hypertension (%)	13 (28.89)	18 (33.96)	0.290	0.590
Diabetes (%)	3 (6.67)	4 (7.55)	0.028	0.866
hyperlipaemia (%)	29 (64.44)	30 (56.60)	0.624	0.429
TG (mmol/L)	1.39 ± 0.44	1.47 ± 0.41	−0.954	0.342
TC (mmol/L)	4.00 ± 1.28	4.15 ± 1.06	−0.613	0.541
HDL-C (mmol/L)	1.09 ± 0.32	1.19 ± 0.39	−1.364	0.176
LDL-C (mmol/L)	2.47 ± 0.70	2.65 ± 0.94	−1.008	0.280
Tei index	0.58 (0.51, 0.72)	0.44 (0.35, 0.52)	2.550	<0.001
Carotid IMT (mm)	1.11 (0.89, 1.26)	0.72 (0.62, 0.80)	3.109	<0.001
Crouse plaque score	1.83 (1.04, 2.56)	0.90 (0.77, 1.09)	2.561	<0.001

### 3.2 Analysis of coronary heart disease-related risk factors

Using the presence of severe calcification as the dependent variable, univariate and multivariate binary Logistic regression analyses were conducted to identify independent risk factors for severe calcification among cardiac and cervical ultrasound parameters. As shown in Table 2, the Tei index, carotid IMT, and Crouse plaque score emerged as significant risk factors for severe calcification (All *P* < 0.05). Specifically, an increase in any of these parameters significantly elevated the risk of severe calcification. After adjusting for confounding factors, the Tei index, carotid IMT, and Crouse plaque score remained independent risk factors for severe calcification (All *P* < 0.05) (Table 3). Notably, a unit increase in the Tei index, carotid IMT, or Crouse plaque score independently increased the risk of severe calcification by 72.061-fold, 6.341-fold, or 1.634-fold, respectively. Given the restricted physiological range of the Tei index (approximately 0.2–0.8 in clinical practice), the large OR for a 1-unit change should be interpreted with caution; it primarily signifies a very strong statistical association, whereas a more clinically relevant effect size can be inferred from the unitless regression coefficient (B) or the marked intergroup difference shown in Table 1.

**Table 2:** Analysis of coronary heart disease-related risk factors in cardiac and cervical vascular ultrasound parameters (unadjusted).

Parameters	B	SEM	Waldx^2^	*P* value	OR	95%CI	
						Lower limit	Upper limit
Tei factor	7.193	1.715	17.593	<0.001	1,330.390	46.151	38,350.631
IMT	3.391	0.832	16.625	<0.001	29.691	5.818	151.528
Plaque score	1.767	0.408	18.736	<0.001	5.853	2.630	13.027

**Table 3:** Analysis of risk factors related to coronary heart disease in color Doppler ultrasound parameters of heart and neck vessels (correction factors).

Parameters	B	SEM	Waldx^2^	*P* value	OR	95%CI	
						Lower limit	Upper limit
Tei factor	4.291	1.996	4.624	0.032	73.061	1.462	3,650.248
IMT	1.993	0.898	4.925	0.026	7.341	1.262	42.698
Plaque Score	0.969	0.466	4.316	0.038	2.634	1.056	6.570

### 3.3 Analysis of ultrasound parameters across different numbers of severely calcified coronary arteries

Differences in cardiac and cervical vascular ultrasound parameters across varying numbers of severely calcified coronary arteries were assessed using one-way ANOVA. As presented in Table 4, statistically significant differences were observed in the Tei index, carotid IMT, and Crouse plaque score across different numbers of calcified vessels (all *P* < 0.015). *Post-hoc* comparisons using the LSD test revealed that patients with multi-vessel severe calcification had significantly higher Tei indices (*P* < 0.01, ##), carotid IMTs (*P* < 0.01, ##), and Crouse plaque scores (*P* < 0.01, ##) compare to those with single-vessel disease. Additionally, patients with bi-vessel severe calcification had significantly higher carotid IMTs and Crouse plaque scores than those with single-vessel disease (both *P* < 0.05), while no significant difference was found in the Tei index (*P* > 0.05). Although patients with multi-vessel severe calcification exhibited higher Tei indices, carotid IMTs, and Crouse plaque scores than those with bi-vessel calcification, these differences were not statistically significant (All *P* > 0.05). Further analysis of the correlation between ultrasound parameters and the number of severely calcified coronary arteries is presented in Table 5. Spearman's correlation analysis revealed significant positive correlations between the Tei index, carotid IMT, Crouse plaque score, and the number of severely calcified coronary arteries (*r* > 0, *P* < 0.05), indicating that higher values of these parameters were associated with a greater number of severely calcified vessels.

**Table 4:** Comparative analysis of cardiac and cervical vascular ultrasound parameters across different numbers of severely calcified coronary arteries.

Parameters	*N*	Tei factor	Carotid IMT (mm）	Crouse plaque score
Single branch severe calcification	16	0.52 ± 0.18^##^	0.91 ± 0.28*^,^^##^	1.36 ± 0.78*^,^^##^
Double branches of severe calcification	16	0.62 ± 0.14	1.14 ± 0.25	1.99 ± 0.82
Multiple severe calcifications	13	0.71 ± 0.15	1.22 ± 0.28	2.22 ± 0.81
F value		9.156	10.995	10.135
*P* value		0.011	0.008	0.015

* *P* < 0.05 *vs.* Double branches of severe calcification. ^##^ *P* < 0.01 *vs.* Multiple severe calcifications.

**Table 5:** Correlation analysis between color Doppler ultrasound parameters of heart and neck vessels and the number of stenosed coronary vessel.

Parameters	Number of stenosed coronary vessel		
	*r*	OR (95% CI)	*P* Value
Carotid IMT (mm）	0.453	(0.176, 0.664)	0.002
Crouseplaque score	0.429	(0.147, 0.647)	0.003
Tei index	0.440	(0.159, 0.655)	0.003

### 3.4 Diagnostic value of cardiac and cervical vascular ultrasound parameters in coronary heart disease

**Figure 1:**
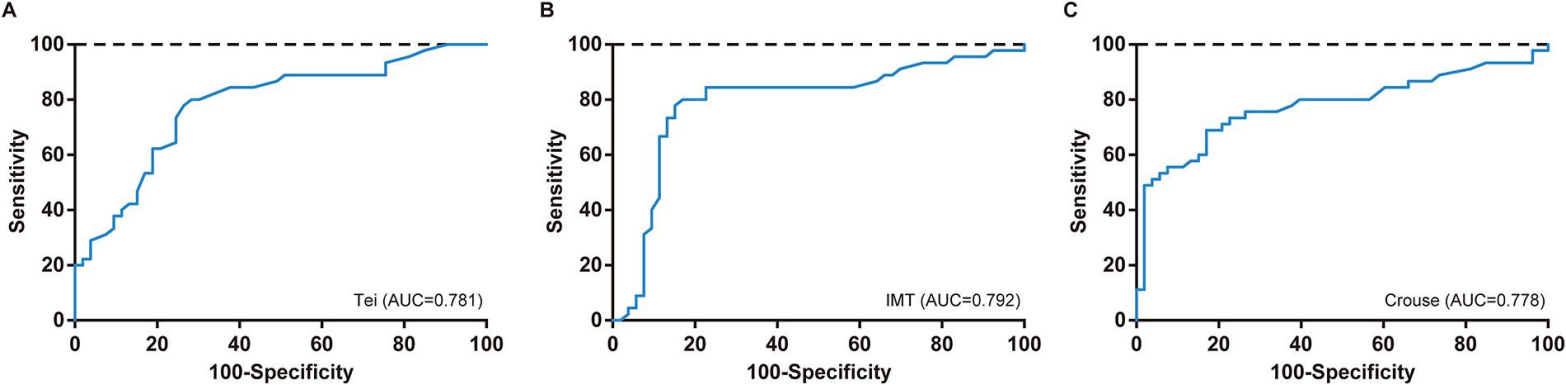
ROC curve of single diagnostic modalities in the diagnosis of severe coronary artery calcification.

**Figure 2:**
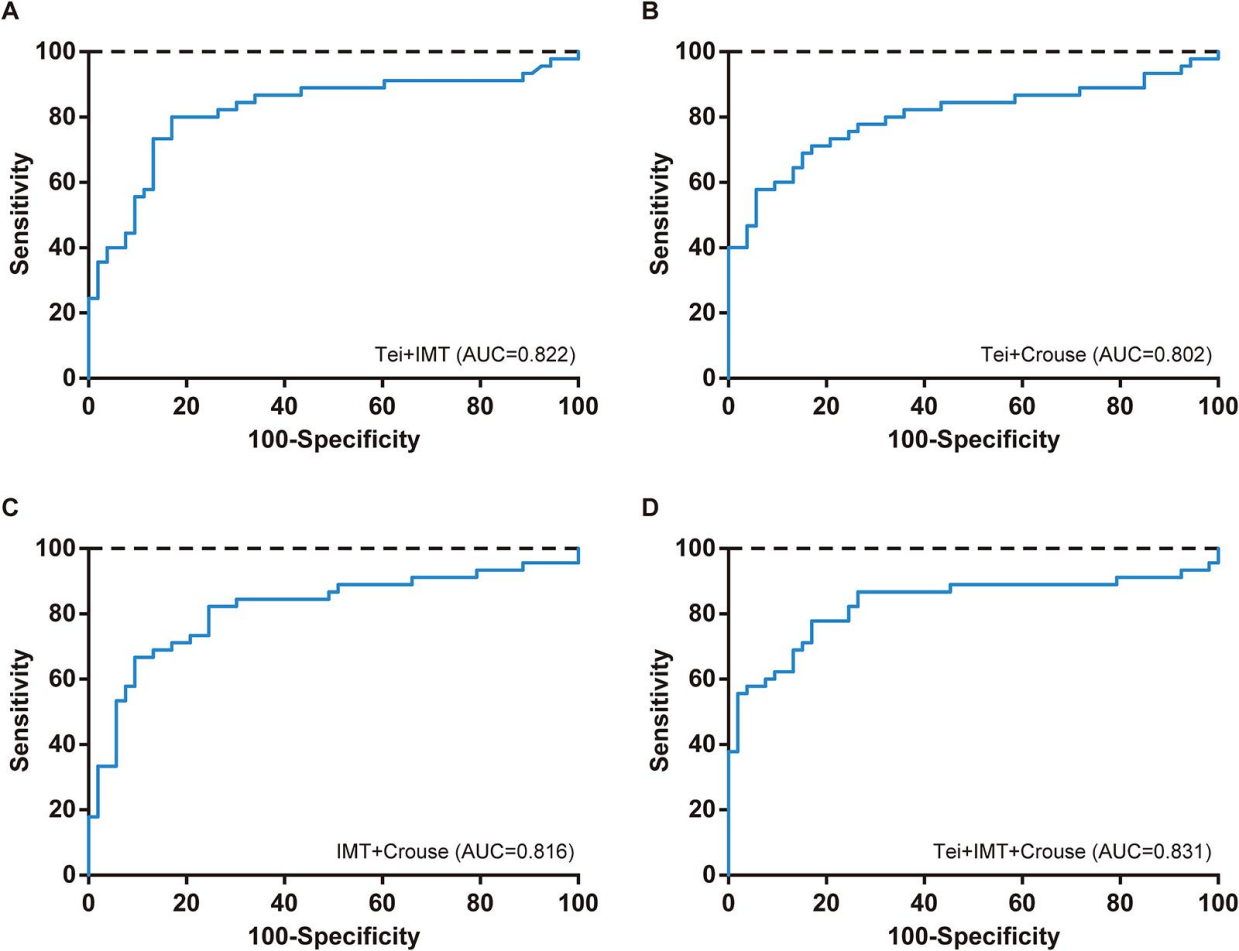
ROC curve of combined diagnosis in the diagnosis of severe coronary artery calcification.

ROC analysis was performed to evaluate the diagnostic performance of the Tei index, carotid IMT, and Crouse plaque score, both individually and in combination, for severe coronary artery calcification. As shown in Table 6 and Figure 1, among the individual diagnostic methods, carotid IMT had the highest ROC AUC, indicating its superior diagnostic efficacy for severe coronary artery calcification. Upon selecting appropriate cut-off values, carotid IMT demonstrated a diagnostic sensitivity of 80.0%, identical to that of the Tei index, but slightly higher than the 68.9% sensitivity of the Crouse plaque score. Additionally, the diagnostic specificity of carotid IMT was 83.0%, equal to that of the Crouse plaque score, but slightly higher than the 71.7% specificity of the Tei index. Furthermore, the diagnostic performance of a combined approach utilizing carotid IMT, Crouse plaque score, and Tei index was analyzed using ROC curves. As depicted in Table 6 and Figure 2, the AUCs of all combined diagnostic indicators were higher than those of any individual diagnostic method, suggesting that combined diagnosis significantly enhances the diagnostic efficacy for severe coronary artery calcification. Among the combined indicators, the combination of the Tei index, carotid IMT, and Crouse plaque score yielded the highest AUC of 0.831. These findings suggest that the combined diagnosis of the Tei index, carotid IMT, and Crouse plaque score holds promise as an early diagnostic predictor for severe coronary artery calcification.

**Table 6:** Comparison of individual and combined diagnostic performance of cardiac and cervical vascular ultrasound parameters.

Parameters	AUC (95% CI)	Youden index	Sensitivity (%)	Specificity (%)	*P* value
Tei	0.781 (0.688, 0.874)	0.517	80.0	71.7	<0.001
IMT	0.792 (0.693, 0.891)	0.630	80.0	83.0	<0.001
Crouse	0.778 (0.679, 0.877)	0.519	68.9	83.0	<0.001
Tei + IMT	0.822 (0.731, 0.912)	0.630	80.0	83.0	<0.001
Tei + Crouse	0.802 (0.707, 0.897)	0.541	71.1	83.0	<0.001
IMT + Crouse	0.816 (0.724, 0.907)	0.577	82.2	75.5	<0.001
Tei + IMT + Crouse	0.831 (0.740, 0.923)	0.608	77.8	83.0	<0.001

### 3.5 Predictive value of echocardiographic parameters of heart and cervical vascular ultrasound in the prognosis of severe coronary artery calcification

The predictive value of the Tei index, carotid IMT, and Crouse plaque score in the prognosis of severe coronary artery calcification (CAC) was assessed through Cox regression analysis. During the follow-up period, MACCE occurred in 22 out of 45 patients (48.89%) in the focal severe calcification group. As presented in Table 7, all three parameters—carotid IMT, Crouse plaque score, and Tei index—were significant predictors of cardiovascular events in the prognosis of severe CAC in univariate analysis (all *P* < 0.001). Specifically, an unadjusted increase of 1 unit in Tei index, carotid IMT, or Crouse plaque score significantly elevated the risk of cardiovascular events by 447.941, 22.843, or 4.408 times, respectively. Additionally, in the multivariate Cox proportional hazards model analysis, after adjusting for other relevant factors, Tei index, carotid IMT, and Crouse plaque score remained significant predictors of recurrent cardiovascular events in prognosis (all *P* < 0.05). Notably, each increment of 1 unit in these parameters independently and significantly raised the risk of cardiovascular events by 48.056, 8.454, or 2.097 times, respectively.

**Table 7:** Cox regression analysis of echocardiographic parameters of heart and cervical vascular ultrasound in coronary heart disease recurrence.

Variable	Univariable Analysis			Multivariable Analysis		
	Unadjusted HR	95% CI	*P*	Adjusted HR	95% CI	*P*
Tei	448.941	32.873–6,131.048	<0.001	49.056	1.384–1,738.301	0.032
IMT	22.843	3.879–134.534	<0.001	9.454	1.176–76.024	0.035
Crouse	5.408	2.624–11.148	<0.001	3.097	1.305–7.345	0.010

**Figure 3:**
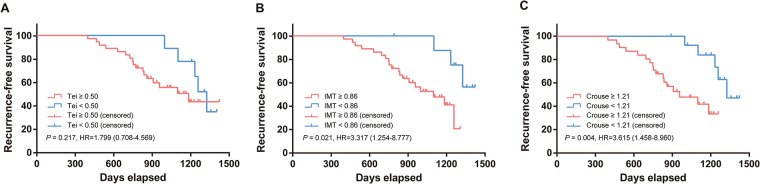
Predictive value of echocardiographic parameters of heart and cervical vascular ultrasound in coronary heart disease recurrence.

Based on the results in Table 6, the diagnostic performance for severe CAC was optimal when carotid IMT, Crouse plaque score, and Tei index reached their respective cut-off values. Therefore, using these cut-off values as thresholds, we further analyzed the predictive value of these variables for cardiovascular events in the prognosis of severe CAC. As shown in Figure 3, compared to patients with IMT < 0.86, those with IMT ≥ 0.86 had a significantly increased risk of prognostic cardiovascular events by 2.317 times (*P* < 0.05). Similarly, patients with Crouse ≥ 1.21 had a 2.615-fold higher risk of prognostic cardiovascular events compared to those with Crouse < 1.21 (*P* < 0.01). However, although patients with Tei ≥ 0.50 had a 79.9% increase in risk compared to those with Tei < 0.50, this difference was not statistically significant (*P* > 0.05).

## 4 Discussion

Cardiac color Doppler ultrasound is instrumental in evaluating cardiac function by identifying abnormal patterns and locations of ventricular wall motion, thereby facilitating the assessment of the degree and extent of myocardial ischemia resulting from coronary involvement (29). Research suggests that coronary and carotid atherosclerosis share analogous pathological processes and risk factors, with a strong correlation existing between coronary and carotid artery diseases (16, 17). Studies have demonstrated that IMT serves as an early indicator of atherosclerosis, with a 0.1 mm increase in carotid IMT in patients with CHD increasing the risk of acute myocardial infarction by approximately 11% (30, 31). Furthermore, the Crouse plaque score is emerging as a significant marker for coronary atherosclerosis diagnosis (32). These findings underscore the potential utility of the Tei index, carotid IMT, and Crouse plaque score in diagnosing severe CAC. Our study indicate show that the Tei index, carotid IMT, and Crouse plaque score were significantly elevated in patients with severe CAC compared to controls, suggesting that echocardiography and carotid ultrasound are valuable tools in the diagnosis of severe CAC.

Further logistic regression analysis revealed that Tei index, carotid IMT, and Crouse plaque score were independent risk factors for severe calcification in elderly CHD patients. The risk of severe calcification increased with higher Tei index, carotid IMT, and Crouse plaque score. This can be attributed to CAC-induced coronary vascular wall stiffening, impaired vasomotor function, and reduced myocardial perfusion. These factors further elevate the Tei index. Moreover, both carotid and coronary arteries are muscular arteries, with carotid plaques commonly found at the carotid bifurcation, likely due to turbulent blood flow and uneven velocity, which easily causes vascular endothelial damage. Coronary atherosclerosis leads to the formation of mural thrombi or intimal plaques, and CAC is a direct marker of coronary atherosclerosis. Thus, carotid atherosclerosis can serve as a window for assessing severe CAC. Additionally, correlation analysis showed that Tei index, carotid IMT, and Crouse plaque score were significantly positively correlated with the number of diseased vessels in severe CAC in elderly CHD patients, suggesting their potential as early diagnostic markers for CAC vessel involvement, guiding subsequent interventional treatment plans.

CCTA is widely used in CAC diagnosis due to its simplicity, high accuracy, and non-invasiveness. Dual-source dual-energy CT imaging (DSCT), photon-counting CT (PCCT), and CT-derived fractional flow reserve (CT-FFR), all based on CCTA, are effective tools for coronary calcification diagnosis (33–35). However, these methods still face numerous clinical validation and application challenges. DSCT instruments are expensive, with costs approximately 25% higher than equivalent single-energy CT, and the cost of their reconstruction and post-processing systems is also substantially increased (33). Moreover, DSCT requires highly skilled operators for scanning and post-processing, necessitating systematic training and extensive experience (33). Additionally, there is a possibility of mismatch between the two energy datasets in DSCT (36). While PCCT technology offers higher spatial resolution and more pronounced density contrast than DSCT (35), its image and post-processing data are voluminous, and its clinical application remains in the laboratory stage, requiring further exploration (37). The diagnostic efficacy of CT-FFR for CAC is influenced by different algorithms and CCTA image quality (38). Our study used ROC analysis to evaluate the diagnostic performance of echocardiographic and carotid ultrasound parameters for severe CAC in elderly CHD patients. Among individual diagnostic modalities, carotid IMT demonstrated the highest diagnostic performance for severe CAC, with sensitivity and specificity of 80.0% and 83.0%, respectively. Furthermore, the combined diagnosis of Tei index, carotid IMT, and Crouse plaque score further enhanced the diagnostic performance for severe CAC in elderly CHD patients. These results suggest that the combined diagnosis of Tei index, carotid IMT, and Crouse plaque score has the potential to become an early auxiliary diagnostic method for severe coronary artery calcification.

The interventional treatment for severe coronary artery calcification (CAC) lesions remains a formidable clinical challenge. Currently, rotational atherectomy (RA) is one of the primary interventional strategies for patients with severe CAC (39). Patients with severe CAC often present with various complex comorbidities and are often elderly, leading to a marked increase in the incidence of interventional treatment-related complications and major adverse cardiovascular events (MACEs) (39). Consequently, analyzing the risk factors for long-term major adverse cardio-cerebrovascular events (MACCEs) post-RA in severe CAC patients, constructing predictive models, and assessing the risk of future adverse events hold significant clinical importance. This study, leveraging retrospective cohort data on Tei index, carotid intima-media thickness (IMT), and Crouse plaque score, constructed a predictive model to guide the clinical screening of high-risk patients with severe CAC undergoing RA. Cox regression analysis revealed that Tei index, carotid IMT, and Crouse plaque score were all significant predictors of MACCEs in severe CAC prognosis. Specifically, a unit increase in Tei index, carotid IMT, or Crouse plaque score independently and significantly elevated the risk of MACCEs by 48.056-fold, 8.454-fold, or 2.097-fold, respectively. Furthermore, we stratified elderly patients with severe CAC into test and control sets based on cut-off values of different factors, balanced errors arising from non-experimental factors, and analyzed the risk of MACCEs using Kaplan–Meier survival curves. Results indicated that patients with carotid IMT ≥ 0.86 or Crouse plaque score ≥1.21 had a significantly increased risk of MACCEs, suggesting they are high-risk individuals. Prognostic analysis and predictive modeling for patients with severe CAC undergoing RA can assist clinicians in identifying high-risk patients, formulating personalized treatment and follow-up plans, and ultimately improving patient outcomes.

This study has the following limitations: (1) While consecutive patients meeting the criteria were enrolled to minimize selection bias, the findings from our specialized center may not be fully representative of the broader community or different clinical settings. The results should thus be considered preliminary and hypothesis-generating. With a relatively small cohort and multiple predictor variables included simultaneously, there is an inherent risk that the models may be overfitted. The primary value of these models is hypothesis-generating; their robustness and generalizable predictive performance must be rigorously validated in future, larger prospective studies; (2) All included cases were coronary heart disease patients, limiting the applicability of findings to the general population, necessitating external validation to enhance model generalizability; (3) Our study defined the study group based on a focal calcification threshold (single-vessel CACS ≥ 100) rather than the total Agatston score, which may limit direct comparisons with studies using the latter classification. However, this approach allowed us to specifically investigate the biomarkers associated with high-risk, locally severe calcific lesions. Future studies should establish subgroups based on different calcification levels for further exploration; (4) The high-risk thresholds for the Tei index, IMT, and Crouse score were derived from the diagnostic ROC analysis in the same cohort and then applied to predict outcomes. While they effectively stratified risk in our study, these specific cut-off values are exploratory and require external validation in independent, prospective studies to confirm their generalizability and optimize their predictive performance for clinical use.

In conclusion, this study is the first to demonstrate the correlation between Tei index, carotid IMT, Crouse plaque score, and severe CAC in elderly coronary heart disease patients. The combined use of these three indicators exhibits high diagnostic performance for severe CAC in this patient population, providing reliable guidance for early screening. Additionally, Tei index, carotid IMT, and Crouse plaque score can serve as predictive indicators for MACCEs post-RA in elderly patients with severe CAC.

## Data Availability

The original contributions presented in the study are included in the article/Supplementary Material, further inquiries can be directed to the corresponding author.
